# cAMP and the Fibrous Sheath Protein CABYR (Ca^2+^-Binding Tyrosine-Phosphorylation-Regulated Protein) Is Required for 4D Sperm Movement

**DOI:** 10.3390/ijms231810607

**Published:** 2022-09-13

**Authors:** Linda Frintrop, Caroline Wiesehöfer, Aura Stoskus, Gero Hilken, Marko Dubicanac, Nicola Edith von Ostau, Sebastian Rode, Jens Elgeti, Jaroslaw Thomas Dankert, Gunther Wennemuth

**Affiliations:** 1 Institute of Anatomy, Rostock University Medical Center, 18057 Rostock, Germany; 2 Institute of Anatomy, Department of Anatomy, University Duisburg-Essen, 47057 Essen, Germany; 3 Central Animal Laboratory, University Hospital Essen, 47057 Essen, Germany; 4 Department of Urology, University Hospital Essen, 45147 Essen, Germany; 5 Theoretical Soft Matter and Biophysics, Institute of Complex Systems and Institute for Advanced Simulation, Forschungszentrum Jülich, 52425 Jülich, Germany

**Keywords:** digital holographic microscopy, spermatozoa, detergent-extracted model, digital-holographic microscopy, Ca^2+^-binding tyrosine-phosphorylation-regulated protein (CABYR)

## Abstract

A new life starts with successful fertilization whereby one sperm from a pool of millions fertilizes the oocyte. Sperm motility is one key factor for this selection process, which depends on a coordinated flagellar movement. The flagellar beat cycle is regulated by Ca^2+^ entry via CatSper, cAMP, Mg^2+^, ADP and ATP. This study characterizes the effects of these parameters for 4D sperm motility, especially for flagellar movement and the conserved clockwise (CW) path chirality of murine sperm. Therefore, we use detergent-extracted mouse sperm and digital holographic microscopy (DHM) to show that a balanced ratio of ATP to Mg^2+^ in addition with 18 µM cAMP and 1 mM ADP is necessary for controlled flagellar movement, induction of rolling along the long axis and CW path chirality. Rolling along the sperm’s long axis, a proposed mechanism for sperm selection, is absent in sea urchin sperm, lacking flagellar fibrous sheath (FS) and outer-dense fibers (ODFs). In sperm lacking CABYR, a Ca^2+^-binding tyrosine-phosphorylation regulated protein located in the FS, the swim path chirality is preserved. We conclude that specific concentrations of ATP, ADP, cAMP and Mg^2+^ as well as a functional CABYR play an important role for sperm motility especially for path chirality.

## 1. Introduction

In 20% infertility of couples caused by the male factor [1]. The primary causes of male infertility are defects in sperm motility and capacitation. Capacitated sperm are capable of hyperactivated motility, a powerful, asymmetric, whip-like motion of the tail [2,3,4,5,6], which enables sperm to free themselves from the oviductal epithelium and breaches the zona pellucida to fuse with the egg [7,8]. Sperm motility is regulated by several metabolic pathways like Ca^2+^ and the cyclic adenosine monophosphate (cAMP)-dependent protein kinase pathway [3,6,9,10,11,12,13,14]. Ca^2+^ entry into the sperm is predominantly enabled by CatSper, a voltage-dependent Ca^2+^ channel, located along the principal piece of flagellum and activated by alkalinization [15,16,17,18,19,20,21]. Inactivation of different ion channels in mice, especially knockout of the CatSper channel leads to a change in the intracellular ionic milieu, which causes infertility [22,23]. Therefore, intracellular Ca^2+^ increase is crucial for capacitation, hyperactivation, acrosome reaction and fertilization.

Beside Ca^2+^, other factors like Mg^2+^, ADP, ATP and cAMP are regulators of the flagellar beat cycle [24,25,26,27,28,29,30]. The function of different intracellular concentrations of these components was analyzed in a detergent-extracted model, in which the plasma membrane of sperm is removed by using non-ionic detergents [24,25,31]. This is a useful tool to study the regulatory function of different factors for motility of flagella. Lindemann et al. proposed that these detergent-extracted sperm need a balanced ratio of ATP and Mg^2+^ for maintaining a measurable beat cycle, otherwise the sperm are only jittering [25]. A too large excess of one of these substances led to a loss of beat cycle.

The few sperm reaching the fertilization site [32,33] are chosen by a defined selection process based on different navigation mechanisms like rheotaxis [33,34], chemotaxis [35,36], thermotaxis [37] or episodic rolling of mouse sperm along their long axis [38]. Sperm without rolling behavior are swimming circles [38,39], underlining the importance of episodic rolling for linear swimming trajectories and therefore for a successful reaching the oocyte. Our group demonstrated that rolling around the long axis in murine sperm is due to changes of sidedness of the head [38]. This rolling behavior in linear swimming sperm displays an alternating clockwise (CW) and counter clockwise (CCW) pattern for 180° rotations [39]. Also, rheological properties are essential to switch from one swimming pattern to a different one in bovine sperm. Surface exploration leads to circular swimming sperm while wall-dependent navigation and rheotaxis lead to linear swimming sperm with rolling [40]. In addition, also microtubule glycylation accompanied with abnormal conformations of the dynein arms impaired murine linear swimming patterns indicating their importance in rolling behavior [41]. A role of the CatSper channel and its regulatory function in intracellular Ca^2+^ homeostasis for rolling along the long axis could be disproven [42]. In contrast, by the use of digital holographic microscopy (DHM) we have shown, that trajectories of non-capacitated as well as capacitated murine wildtype (wt) sperm have a conserved CW chirality [39,43], which is lost in sperm lacking either the entire CatSper channel or its Ca^2+^ sensor EF-hand calcium-binding domain-containing protein 9 (EFCAB9) [43]. Additionally, contact of capacitated sperm to the zona pellucida glycoprotein 2 (ZP2) led to an elimination of the CW path chirality indicating an important role of Ca^2+^-homeostasis for orchestrating CW swimming path chirality [43].

The aim of this work is to investigate the 4D sperm movement, in particular the rolling of sperm along their longitudinal axis and the chirality of their swimming trajectory with respect to regulatory factors. For this purpose, the effect of Ca^2+^, cAMP, Mg^2+^, ADP and ATP, as well as stabilizing structures in the sperm flagellum such as the outer-dense fibers (ODF) and the fibrous sheath (FS) [44] are investigated using sea urchin sperm and sperm of animals with a mutation of the *Cabyr* (Ca^2+^-binding tyrosine-phosphorylation regulated protein) gen. CABYR is localized in the principle piece of the sperm FS and associated with calcium signaling pathways [45,46].

## 2. Results

### 2.1. Defined cAMP and ADP Concentrations Are Necessary for Murine Chiral Movement

**Figure 1:**
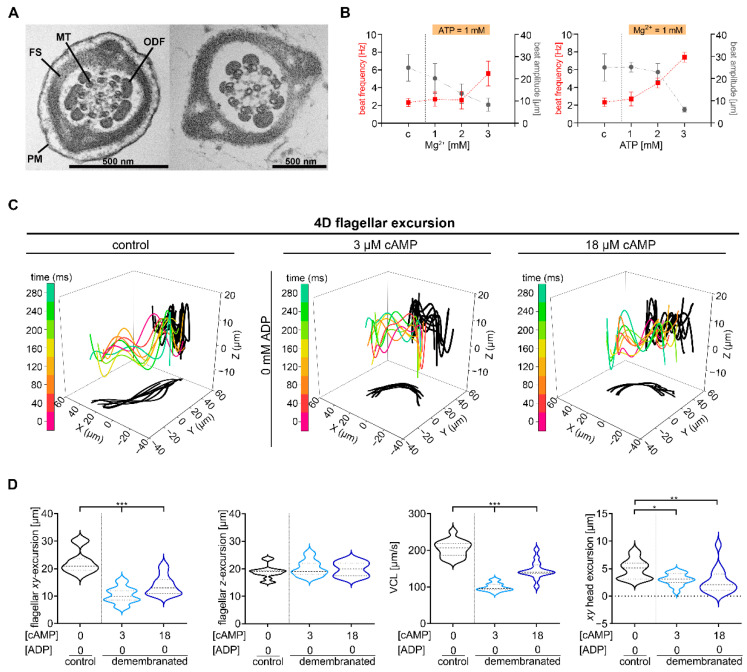
4D motility of demembranated wt sperm. (**A**) Representative transmission electron microscopy cross sections of flagella mid-pieces of membranated (left) and demembranated (right) sperm. MT—microtubules, FS—fiber sheath, PM—plasma membrane, ODF—outer dense fiber. Scale bar = 500 nm. (**B**) Analysis of beat frequency and beat amplitude of demembranated mouse sperm with 1 mM ATP and different Mg^2+^ concentrations (1–3 mM) (left) or with 1 mM Mg^2+^ and different ATP concentrations (1–3 mM) (right). An increase in ATP or Mg^2+^ concentration results in an increase in beat frequency and a decrease in the beat amplitude compared to membrane intact control sperm (c). mean ± SEM; N = 4, n = 12. (**C**) 4D waveform analysis of intact sperm (first panel) compared with demembranated sperm analyzed without ADP but with increasing cAMP concentrations (0, 3, 18 µM). Traces of a flagellum in 3D at different time points (0, 40, …, 280 ms) are color-coded and the projections onto the *XY*-and *XZ*-plane are shadowed in black. (**D**) Statistical analysis of *XY*-(first panel) and *Z*-excursions of flagella (second panel), 3D curvilinear velocity (VCL) (third panel) and lateral head displacement (forth panel) of intact and demembranated sperm in the presence of different cAMP and ADP concentrations (color-coded). N = 4, n = 15, * *p* < 0.05, ** *p* < 0.01, *** *p* < 0.001, median: thick dashed lines; interquartile range: thin dashed lines.

Different intracellular mediators like cAMP, calcium and pH regulate flagellar sperm movement and are essential for capacitation, a process necessary for a successful fertilization. Analysis with Triton X-100-extracted, Mg^2+^-ATP-reactivated bull sperm shows a regulatory function of ATP, ADP and Mg^2+^ for the beat cycle [24,25,26]. To identify which factors are required for the chiral movement of mouse sperm and whether the influence of these factors is concentration-dependent, the detergent-extracted model was applied to wt mouse sperm (NMRI mice, Charles River) in analogy to Lindemann and Gibbons [28]. In the principal piece of mammalian sperm flagella the axoneme is surrounded by ODFs, mitochondria, a FS and a plasma membrane [44] (Figure 1A, left). Successful demembranation of mouse sperm flagella was shown by transmission electron microscopy (TEM) in sperm extracted with 0.1% Triton X-100 (Figure 1A, right). A stable beat cycle of bull sperm depends on a ATP-Mg^2+^- balance [25]. Local perfusion of sperm with increasing ATP or Mg^2+^ concentrations were performed to describe the role of ATP and Mg^2+^ for flagellar movement of demembranated mouse sperm (Figure 1B). Demembranated sperm analyzed under a balanced ATP to Mg^2+^ concentration show a comparable beat amplitude (~23 µm) and frequency (3 Hz) as intact sperm (beat amplitude: ~25 µm; beat frequency: 2 Hz). With increasing ATP or Mg^2+^ concentrations the beat amplitude decreases and the beat frequency increases.

**Figure 2:**
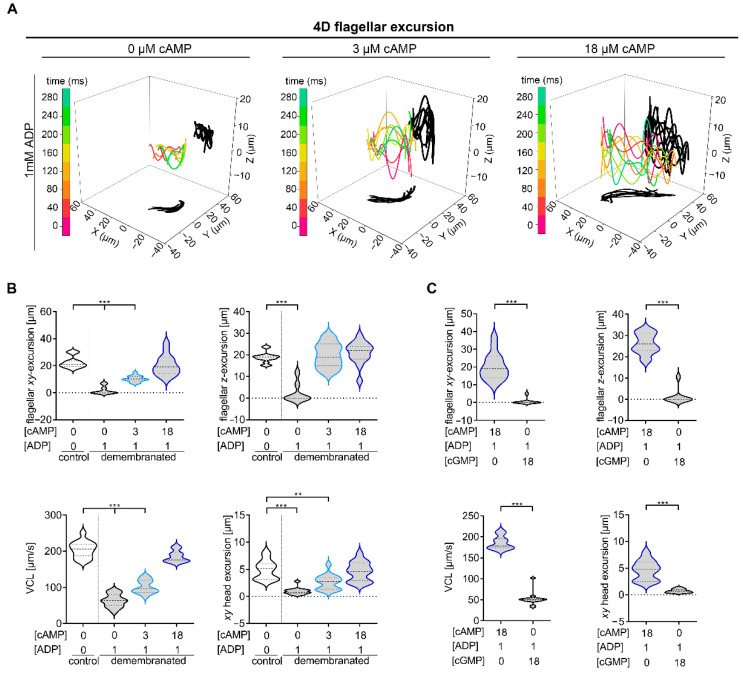
4D motility of demembranated wt sperm can be recovered by adding 18 µM cAMP and 1 mM ADP. (**A**) 4D waveform analysis of demembranated sperm analyzed with 1 mM ADP and 0 cAMP (first panel), 1 mM ADP and 3 µM cAMP (second panel) and 1 mM ADP and 18 µM cAMP (third panel). Color-coded lines are traces of a flagellum in 3D at different time points (0, 40, …, 280 ms) and the projections onto the *XY*-and *XZ*-plane are shadowed in black. (**B**) Comparative statistical analysis of flagellar *XY*-(upper panel, left) and *Z*-excursions (upper panel, right), 3D curvilinear velocity (lower panel, left) and lateral head displacement (lower panel, right) of demembranated sperm measured with 1 mM ADP and 0 µM cAMP or in the presence of 3 µM or 18 µM cAMP. (**C**) Comparative statistical analysis of flagellar *XY*-(upper panel, left) and *Z*-excursions (upper panel, right), 3D curvilinear velocity (lower panel, left) and lateral head displacement (lower panel, right) of demembranated sperm measured in the presence of cAMP or cGMP. cGMP does not recover sperm motility in demembranated sperm. N = 4, n = 15, ** *p* < 0.01, *** *p* < 0.001, median: thick dashed lines; interquartile range: thin dashed lines.

To examine the effect of cAMP and ADP on chiral movement, we used DHM obtained from samples of sperm with intact membrane as a control and demembranated mouse sperm that were observed under different cAMP and ADP conditions (Figure 1 and Figure 2) (cAMP and ADP combinations used: (1) 3 µM cAMP, 0 mM ADP; (2) 18 µM cAMP, 0 mM ADP; (3) 0 µM cAMP, 1 mM ADP; (4) 3 µM cAMP, 1 mM ADP; (5) 18 µM cAMP, 1 mM ADP). Demembranated sperm analyzed under condition 1 and 2 show a significant decrease of flagellar *XY*-excursions (1: median 10 µm, 2: median 13 µm), of curvilinear velocity (VCL) (1: median 96 µm/s, 2: median 141 µm/s) and lateral head displacement (1: median 3.1 µm, 2: median 2.1 µm) in comparison to intact sperm (flagellar *XY*-excursion: median 22.5 µm, VCL: median 205.0 µm/s, lateral head displacement: median 5.0 µm) (Figure 1C,D). In contrast, flagellar *Z*-excursions were not significantly decreased in demembranated sperm under conditions used (1: median 20.3 µm, 2: median 19.9 µm) compared to intact sperm (median 18.9 µm).

Next, we investigated whether ADP alone or a combination of ADP and cAMP leads to 4D movement like that of intact sperm. For this purpose, digital holographic recordings of demembranated sperm measured under condition 3–5 ((3) 0 µM cAMP, 1 mM ADP; (4) 3 µM cAMP, 1 mM ADP; (5) 18 µM cAMP, 1 mM ADP) were performed (Figure 2).

4D movement of demembranated sperm measured under condition 3 is significantly decreased (flagellar *XY*-excursion: median 0 µm, flagellar *Z*-excursion: median 0 µm, VCL: median 63.43 µm/s, lateral head displacement: median 0.77 µm) in comparison to intact sperm (flagellar *XY*-excursion: median 22.5 µm, flagellar *Z*-excursion: median 18.9 µm, VCL: median 205.0 µm/s, lateral head displacement: median 5.0 µm) (Figure 2A,B). Sperm analyzed under condition 4 also showed a significant decrease in sperm movement, with the exception of flagellar movement in the Z-plane (median 19 µm) (Figure 2A,B). Only sperm measured with 18 µM cAMP and 1 mM ADP (flagellar *XY*-excursion: median 20.3 µm, flagellar *Z*-excursion: median 21 µm, VCL: median 185.6 µm/s, lateral head displacement: median 4.6 µm) display a similar movement like intact sperm. These results indicate that defined conditions of cAMP (18 µM) in combination with ADP (1 mM) are required for the necessary flagellar beat movement and sperm motility in all three dimensions.

To exclude that the cyclic structure of cAMP has unspecific effects on sperm motility, we performed equivalent 4D analysis with demembranated sperm prepared in buffer with 18 µM cGMP instead of 18 µM cAMP (Figure 2C). Demembranated sperm measured in the presence of 18 µM cAMP and 1 mM ADP, which showed comparable movement to the membranated sperm, served as control. A significant reduction of all analyzed sperm motility parameters could be detected for demembranated sperm measured in buffer with cGMP, indicating the specificity of cAMP effects on sperm motility.

### 2.2. Rolling along the Long Axis and Conserved CW Path Chirality of Sperm Depends on ADP and cAMP

Factors of the extracellular milieu that may influence sperm selection by creating diversity in sperm swimming behavior include modifications in response to fluid flow (rheotaxis) [33,34], temperature (thermotaxis) [37], and in vitro observed rolling of sperm along their longitudinal axis with transient attachment to structures of the environment [38]. For murine sperm the orientation of the asymmetric head can be characterized as right-cheek (RCh) or left-cheek (LCh) [39]. During rolling along their long axis sperm show alternating light flashes due to reflection of light on the sperm head. Thus, the rolling of linear swimming sperm is analyzed through the transient increase in intensity of light while changing head sides from RCh to LCh and vice versa.

**Figure 3:**
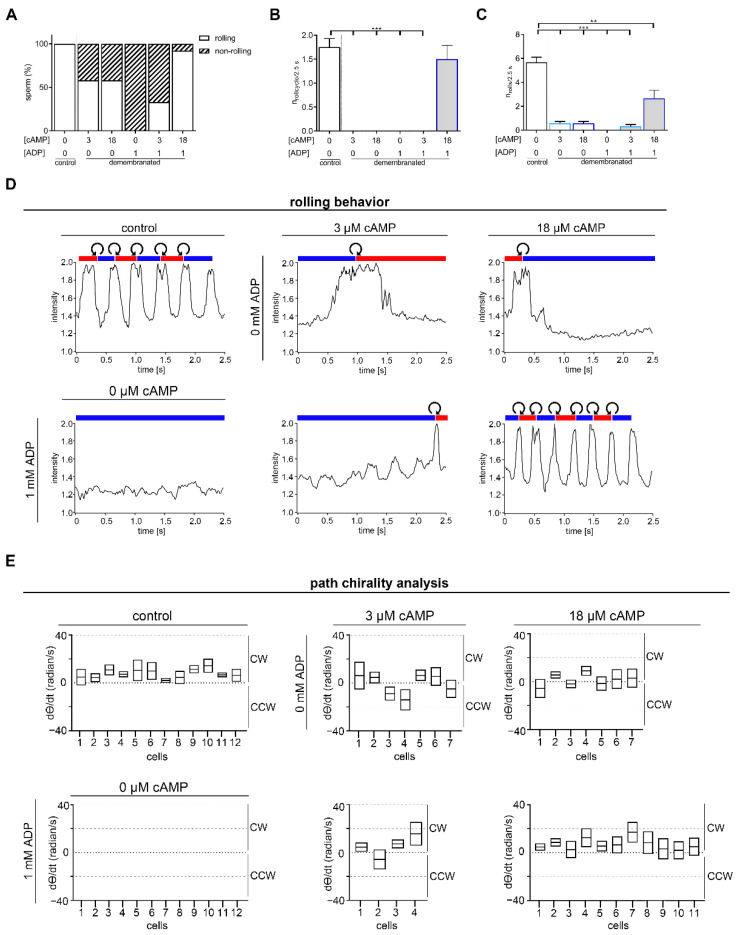
Rolling of sperm requires cAMP and ADP. (**A**–**C**) Statistical analysis of demembranated murine sperm in the presence of defined cAMP and ADP concentrations (color-coded as in Figure 1) compared to membrane intact sperm. (**A**) Percentage of rolling sperm, (**B**) number of rolling cycles during a 2.5 s recording, where a rolling cycle is defined as RCh-LCh-RCh or vice versa, and (**C**) the number of rolling movements characterized by an increase in intensity of light reflected from the head analyzed. (**D**) Analysis of rolling behavior along the long axis of intact sperm (upper panel, left) and of demembranated sperm under different cAMP and ADP concentrations (upper and lower panels) by measuring the oscillating light intensity which scattered from the sperm head. Red and blue bars indicate periods of “RCh” and “LCh” orientation for the sperm head. The short white areas represent an indeterminate orientation and the curved arrows indicate CW or CCW rolling. (**E**) Path chirality analysis using averaged Procrustes alignments. Positive *dθ/dt* values indicate a CW and negative values a CCW chirality. Depending on the different rolling behavior of demembranated sperm due to different cAMP and ADP concentration the number of analyzed sperm varied. N = 4, n = 12, mean ± SEM; ** *p* < 0.01, *** *p* < 0.001.

Like membrane intact sperm, demembranated sperm with a linear swimming trajectory display a rolling behavior including oscillating intensity of light scattered from the rolling head (Figure 3). To test if a dose-dependent concentration or a defined combination of cAMP and ADP is essential for rolling, demembranated sperm were analyzed under the same conditions as in Figure 1. In general, the percentage of rolling sperm is decreased after demembranation and incubation in different cAMP and ADP concentrations in comparison to intact sperm (Figure 3A). Half of the analyzed demembranated sperm measured in the presence of 3 µM cAMP and 0 mM ADP or 18 µM cAMP and 0 mM ADP role, whereas rolling was completely absent in sperm incubated with ADP w/o cAMP only (Figure 3A). Rolling was regained in 33% of the analyzed sperm measured in the presence of 3 µM cAMP and 1 mM ADP. An almost equal number compared of rolling sperm can be found in intact sperm (100%) and in demembranated sperm incubated in the presence of 18 µM cAMP and 1 mM ADP (91.7%) (Figure 3A). To determine the rolling behavior in more detail, we analyzed the number of full roll cycles, characterized by rolling from RCh to LCh to RCh, in a 2.5 s record (Figure 3B). Only demembranated sperm incubated with 18 µM cAMP and 1 mM ADP have a similar number of roll cycles (1.5 ± 0.8 roll cycles/2.5 s) as control sperm (1.8 ± 0.6 roll cycles/2.5 s) (Figure 3B,C). Even if no complete roll cycles could be identified for all analyzed demembranated sperm, incomplete rolling took place, indicated by light reflections at the sperm head, except for demembranated sperm analyzed in the presence of 0 µM cAMP and 1 mM ADP, indicating that cAMP is required to induce and maintain rolling (Figure 3C). Whereas control sperm show 5 to 6 light reflections (mean 5.6 ± 1.4) (Figure 3C), correlating with ~1.8 full roll cycles (Figure 3B), all examined demembranated sperm show on average a reduced number of light reflections during the 2.5 s recording (Figure 3C). Higher magnification and shorter illumination by using DHM [44] allows the analysis of chirality of rolling indicated by the curved arrows (Figure 3D). Regardless of the presence of the plasma membrane, a consistent alternating rolling pattern for sperm measured in the presence of 18 µM cAMP and 1 mM ADP was identified. All analyzed sperm rolled CCW during the transition from RCh to LCh and CW during the transition from LCh to RCh. The rolling behavior was analyzed independently in blind tests by three experimenters.

Besides rolling along their long axis, we analyzed path chirality of intact sperm and demembranated sperm under the different cAMP and ADP conditions (Figure 3E). To determine path chirality Procrustes analysis [39,43] was performed in Igor Pro^TM^ on sequences of roll-counter-roll cycles. For this purpose, the trajectory was divided into two rolling cycles, where possible. For the demembranated sperm, which rolled only once during the 2.5 s recording, the trajectory was divided into two temporal cycles (50 ms before and after the rolling) and compared with each other. The respective starting *X*- and *Y*- values of each cycle were placed at the origin and averaged. To obtain path chirality, the time derivative of theta (*dθ/dt*, radian/s) was calculated, recording CW or CCW direction of the swimming path [39]. All analyzed intact sperm had a positive mean (*dθ/dt)* value indicating a CW chirality (Figure 3E, upper, left panel). To determine, if a conserved CW path chirality depends on ADP or cAMP concentrations, we also performed Procrustes analysis for demembranated sperm, which show rolling (n_3 µM cAMP_ = 7, n_18 µM cAMP_ = 7, n_3 µM cAMP + 1 mM ADP_ = 4, n_18 µM cAMP + 1 mM ADP_ = 11) (Figure 3E). In contrast to intact sperm, positive and negative values, indicating lost chirality of the swimming path, could be detected for demembranated rolling sperm measured in the presence of 3 µM cAMP (positive value: 4/7 cells), 18 µM cAMP (positive value: 4/7 cells) or 3 µM cAMP with 1 mM ADP (positive value: 3/4 cells). Interestingly, all demembranated rolling sperm analyzed in the presence of 18 µM cAMP and 1 mM ADP condition had a conserved CW path chirality, represented by positive mean (*dθ/dt)* values (Figure 3E, lower, right panel).

### 2.3. Invertebrate Sperm Show No Rolling along Their Long Axis

Sea urchin sperm were used as model for simple structured sperm flagella lacking ODFs and FS since 1972 [47]. With this model we are able to analyze the function of ODFs and FS for 4D sperm movement.

**Figure 4:**
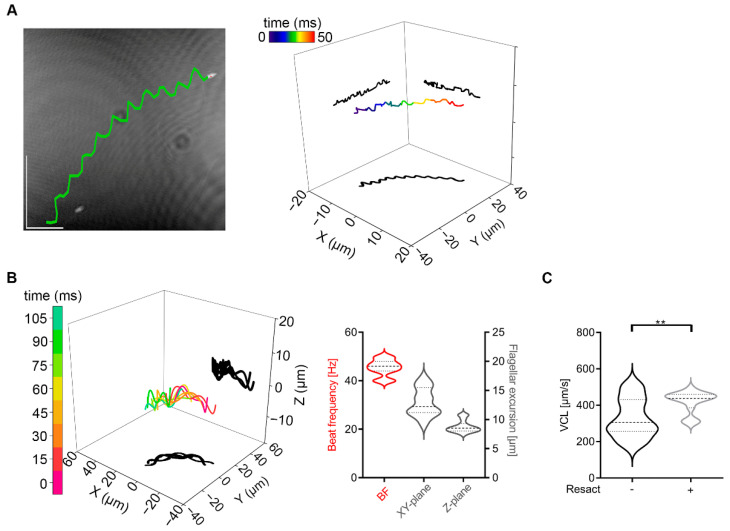
4D motility analyses of sea urchin sperm. (**A**) 50 ms swimming path of a sea urchin sperm overlaid on the reconstructed *XY* projection of the final frame in the 0.5 s holographic record (63X objective) (left) and illustrated in 4D (right). The swimming trajectory is visualized by tracing the head position. (**B**) 4D flagellar waveform analysis whereas the time-lapse trace of a flagellum at 3D position (laboratory-fixed frame of reference xyz) is visualized in color and its projections onto *XY*- and *XZ*-planes are shadowed in black (left). Statistical analysis of beat frequency and flagellar *XY*- and *Z*-excursion (right). (**C**) Statistical analysis of curvilinear velocity. N = 3, n = 15, median: thick dashed lines; interquartile range: thin dashed lines. ** *p* < 0.01.

Non-activated sea urchin sperm swim in circular trajectories by propagating asymmetrical waves down the flagellum [48,49]. We use DHM to confirm these results and to extend the analysis to the third and fourth dimension. In Figure 4A (left) a reconstructed *XY*-plane projection of a sea urchin sperm from the species *Arbacia punctulata* is shown and the trajectory travelled, determined by tracking of the sperm head is superimposed (marked in green). Four-dimensional sperm movement analysis reveals that sea urchin sperm have a circular and planar trajectory (Figure 4A, right). Based on the fact, that sea urchin sperm are nearly 50% smaller in their length than mouse sperm [49], a 63X objective was used to analyze flagellar movement, so that the field of view is smaller than for mouse sperm and the circular trajectory cannot be illustrated to its whole extent (Figure 4A). To further characterize 4D sperm movement of sea urchin sperm, beat frequency and the flagellar movement in *XY*- and *Z*-plane (Figure 4B), and the VCL were determined (Figure 4C). The flagellar movement of sea urchin sperm is characterized by a beat frequency of ~45 Hz (Figure 4B, red), a flagellar *XY*-excursion of 13.0 µm (median) and a flagellar *Z*-excursion of 8.5 µm (median) (Figure 4B, grey), making them smaller as seen in mouse sperm (Figure 1, flagellar *XY*-excursion: median 22.5 µm, flagellar *Z*-excursion: median 18.9 µm). The flagellar *Z*-plane excursions travel as waves down the flagellum (Figure 4B, left) and a VCL of 374 µm/s (median) was detected for sea urchin sperm (Figure 4C).

**Figure 5:**
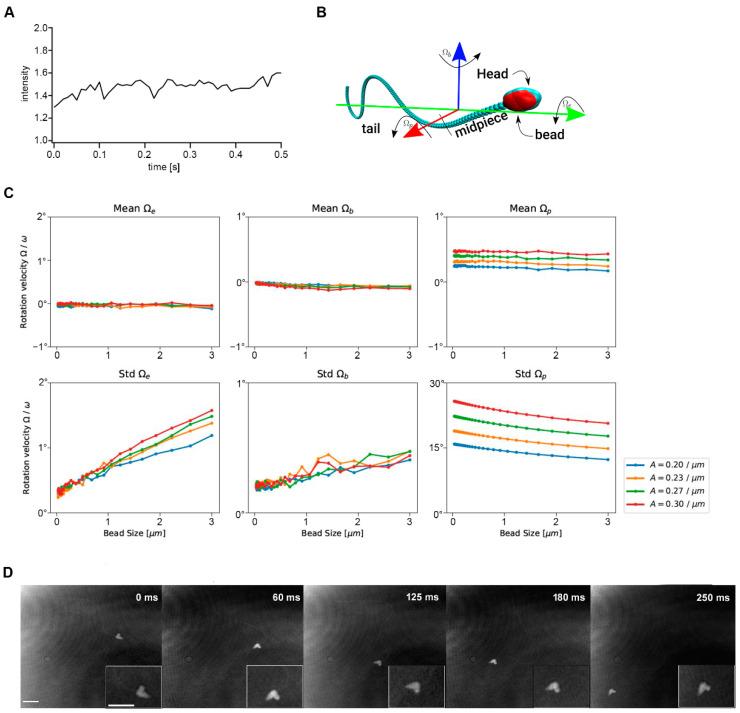
Rolling requires longitudinal stabilizing structures along the flagellum. (**A**) Rolling analysis by measuring the intensity of light scattered from the moving sperm head shows no periodic fluctuations in intensity of reflected light. Based on sea urchin sperm head morphology orientation could not be determined. (**B**) Simulation snapshot of a sperm with an attached bead. Arrows indicate the three main axes of rotation. The different rotation angles (Ω_p_, Ω_b_, Ω_e_) are illustrated. (**C**) Simulation results for the main rotation frequencies, as a function of bead size for various beating amplitudes (A = amplitude of the curvature wave). (**D**) *XY*-projections of a 250 ms holographic record sampled at 198 frames per second (fps) illustrate no rolling of a sea urchin sperm along its long axis by using polystyrene latex beads (0.8 µm mean particle size). Scale bar = 5 µm, N = 3, n = 15.

Sea urchin sperm heads are symmetric with a cone-like shape [49]. Because of this symmetry, different sides of the sperm head cannot be distinguished with light microscopy. As expected, due to the head symmetry, no light flashes could be detected when light intensities were measured (Figure 5A). To visualize possible rolling, we incubated sea urchin sperm with polystyrene latex beads (Merck, 0.8 µm mean particle size) to allow an unspecific binding to the sperm head (Figure 5B,D). During the whole 250 ms holographic recording the latex bead remained at one side of the sea urchin sperm head, indicating no rolling (Supplementary Movie S1). To exclude the possibility, that rolling of sperm is just inhibited by the binding of polystyrene beads due to mechanical interactions, we simulated different conditions mathematically. Figure 5B shows a typical simulation snapshot and the relevant axis of rotation. Different virtual beads of different radius (0–3 µm) at the head of the sperm are simulated (Figure 5C). The usual “up down” wiggling of the sperm head with the beat is visible as a relatively large standard deviation of Ωp. No change in the mean rotation angles is observed, i.e., the attachment of beads up to 3 µm does not lead to an additional net rotation of the sperm cell in any of the three directions (Figure 5C, upper panels). The only significant–despite small—change we observe is a small increase in the standard deviation of Ωe (Figure 5C, lower panels), which means that the bead attachment results in small amplitude (less than a few degrees) wiggling along the long axis of the sperm. Furthermore, no large out-of-plane components of the beat can be seen. In summary, the simulation indicates that attaching beads of up to 3 µm to the sperm head does not lead to experimentally detectable deformations of beat- and swimming pattern.

### 2.4. Cabyr Is Required for Conserved Clockwise Path Chirality of Mouse Sperm

**Figure 6:**
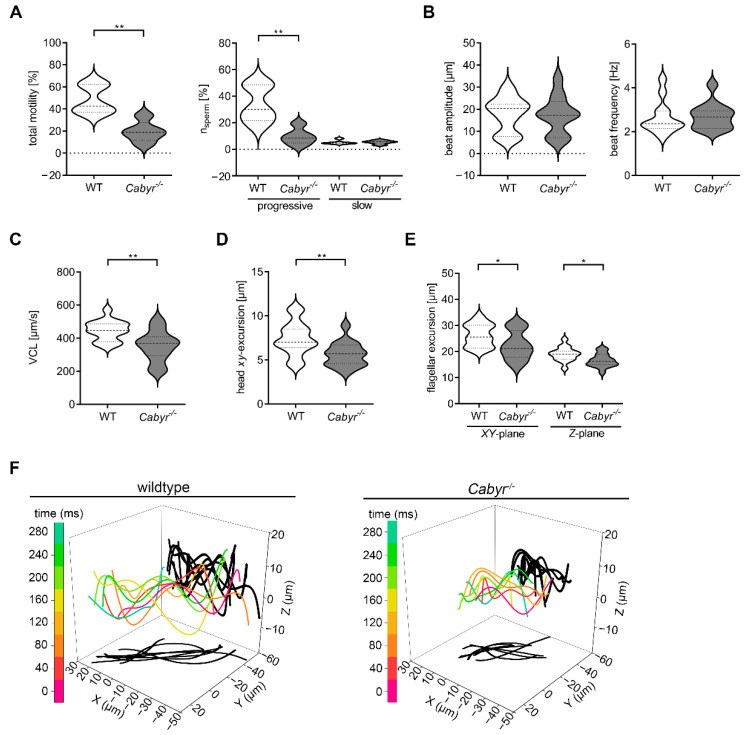
Cabyr–a Ca^2+^ binding tyrosine-phosphorylation-regulated protein—is required for 4D sperm motility. (**A**) Computer-assisted sperm analysis (CASA) reveals decrease of motile sperm in *Cabyr*^−/−^ mice (left), which correlates with a decrease in the number of sperm showing a progressive sperm movement (right). N = 4, n > 800 sperm. (**B**) Flagellar beat amplitude and beat frequency analyzed by CASA are not affected by *Cabyr* knockout. N = 4, n = 15. (**C**) Statistical analysis of curvilinear velocity by DHM. N = 4, n = 15. (**D**) Statistical analysis of lateral head displacement of *Cabyr* knockout in comparison to WT sperm. (**E**) Analysis of flagellar excursion in *XY*- and *Z*-plane in *Cabyr*^−/−^ sperm shows a decrease of flagellar movement. N = 4, n = 15. (**F**) Representative 4D flagellar excursions of wt (left) and *Cabyr^−/−^* (right) sperm in which 3D flagellar excursions at different time points (0, 40, …, 280 ms) are color-coded and its projection onto the *XY*- and *Z*-plane are shadowed in black. * *p* < 0.05, ** *p* < 0.01, median: thick dashed lines; interquartile range: thin dashed lines.

The Ca^2+^-binding tyrosine-phosphorylation-regulated protein (CABYR) is localized to the principal piece of the flagellum in association with the FS [45]. Knockout of *Cabyr* results in subfertility with a defect in sperm motility based on a significant disorganization in the FS [23]. To examine the effect of *Cabyr^−/−^* on the 2D and 4D sperm movement, we used Computer-Assisted-Sperm-Analysis (CASA) and DHM. In general, the motility of *Cabyr^−/−^* sperm is reduced (Figure 6A, left) and the percentage of motile sperm showing a progressive motility is also significantly lower compared to wt sperm (Figure 6A, right). Whereas the beat amplitude and frequency measured by CASA show no differences between *Cabyr^−/−^* and wt (Figure 6B), the lateral head displacement is significantly reduced in sperm of the mutant animals (Figure 6D). To further characterize the movement of *Cabyr^−/−^* sperm, we performed 4D motility analysis, indicating a significant decrease in the VCL (median: wt 446.5 µm/s; *Cabyr^−/−^* 352.9 µm/s) (Figure 6C) and flagellar movement in *XY*- (median: wt 26.2 µm; *Cabyr^−/−^* 22.7 µm) and *Z*-plane (median: wt 19 µm; *Cabyr^−/−^* 16.6 µm) of *Cabyr^−/−^* compared to wt sperm (Figure 6E,F).

**Figure 7:**
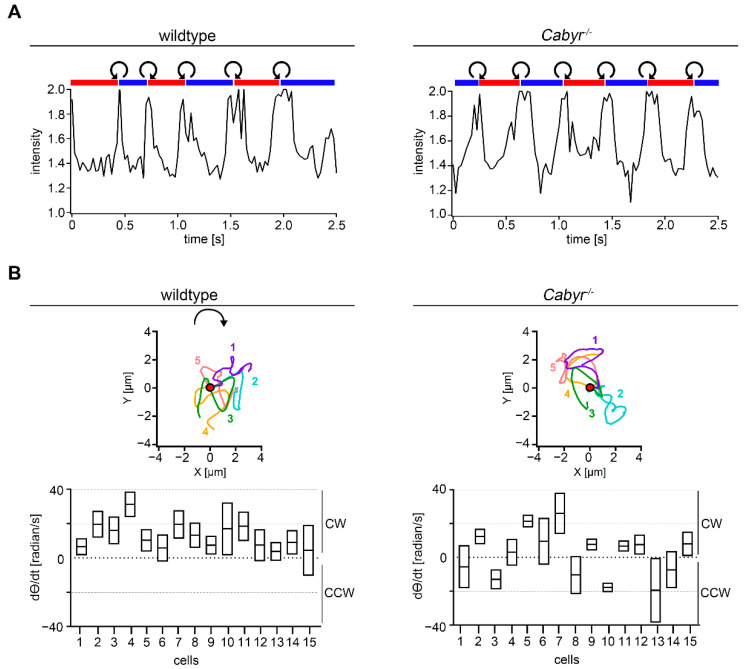
Cabyr facilitates conserved CW path chirality. (**A**) Analysis of rolling behavior along the long axis of wt (left panel) or *Cabyr*^−/−^ (right panel) sperm during a time interval of 2.5 s. Red and blue bars indicate periods of “RCh” and “LCh” orientation for the sperm head. The short white areas represent an indeterminate orientation and the curved arrows indicate CW or CCW rolling. In the diagram, the oscillating light intensity, which is scattered from the sperm head, is shown. *Cabyr*^−/−^ sperm show a comparable rolling behavior as wt sperm. (**B**) Averaged Procrustes alignments of five representative wt (upper, left) or five *Cabyr*^−/−^ sperm (upper, right) translocated to place the initial point at the origin. The curved arrow indicates a CW path chirality analyzed by visual assessment. Determined *dθ/dt* (mean ± SEM) for 15 wt (lower, left) and 15 *Cabyr*^−/−^ sperm (lower, right). Positive *dθ/dt* values indicate a CW and negative values a CCW path chirality. CABYR facilitates conserved CW path chirality.

Next, the effect of *Cabyr^−/−^* on sperm rolling along the long axis by measuring the intensity of the reflected light from the sperm head was analyzed (Figure 7A). For wt and *Cabyr^−/−^* sperm a consistent alternating rolling pattern (RCh-LCh-RCh and vice versa) could be identified. Additionally, CCW rolling took place during the transition from RCh to LCh and CW rolling during the transition from LCh to RCh. These results indicate that the CABYR protein is not necessary for rolling along the long axis. To examine the effect of CABYR on the path chirality of murine sperm, we performed Procrustes analysis. The averaged aligned trajectories of five representative wt sperm (color-coded) show a CW direction of their swimming paths (Figure 7B, upper panel, left). Calculation of the time derivative of theta (*dθ/dt*) (mean ± SEM) of 15 wt sperm demonstrates a conserved CW chirality indicated by a positive mean (*dθ/dt*) value (Figure 7B, lower panel, left). However, *Cabyr^−/−^* sperm did lose their strict CW chirality (Figure 7B, lower panel, right) displayed by both positive (9/15 cells) and negative values (6/15 cells). This indicates that the conserved CW path chirality is lost after *Cabyr*-knockout.

## 3. Discussion

### 3.1. cAMP Plays a Central Role in Regulating Flagellar Movement

Sperm motility is regulated mainly by the Ca^2+^- and cAMP-dependent protein kinase pathways [3,6,9,10,11,12,13,14]. Cellular cAMP is regulated by soluble adenylyl cyclases (sACs), which can be directly activated by bicarbonate and Ca^2+^, acting as a sensor for ATP, Ca^2+^ and bicarbonate [12,14,50,51,52,53]. An essential role of cAMP signaling in sperm development, motility and maturation in the female genital tract is well established [14,54,55]. Microtubule sliding caused by the action of dynein arms leads to the movement of eukaryotic flagella. The regulation of this process is not fully understood, but several regulatory factors like ATP, ADP and cAMP have been indicated [24,25,26,27,28,29,56].

In this study we show that a balanced ratio of ATP and Mg^2+^, as well as a specific combination of cAMP (18 µM) and ADP (1 mM) are necessary for murine flagellar movement in 3D. That a balanced ratio of ATP and Mg^2+^ is necessary for flagellar beating could already have been indicated for bull sperm [24]. Lesich and coworkers showed that the diameter of the axoneme is modified depending on the ratio of ATP to Mg^2+^, resulting in sperm motility defects [24,25]. They postulate that an ATP-Mg^2+^ balance leads to a stable beat cycle. Only in the equilibrium state is a sufficient torque generated by the active dynein. In addition, there is sufficient adhesion of the dyneins to resist deactivation until sufficient curvature is created that allows for a full beat cycle. A regulatory role of ADP for the beat amplitude and frequency was already identified for bull sperm [26]. Excessive amounts or deficiency of any of these substances results in the loss of a controlled beat cycle and disordered motility.

cAMP and its targets regulate a variety of signaling pathways including the increase in intracellular pH [57,58,59] and protein tyrosine phosphorylation [12,53,54,60], which in turn regulates the capacitation process and acrosome reaction [54,61,62]. The bicarbonate-induced increase of cAMP during capacitation activates the cAMP-dependent PKA which phosphorylates axonemal proteins, leading to the induction of flagellar movement [3,14,50,53,60]. Flagellar movement and velocity analysis in this study confirm the central role of cAMP in regulating sperm motility. We could show that cAMP is a precondition for the induction of a lateral head displacement, as well as a flagellar motion in the XY and Z plane resulting in an increase of the swimming speed. In addition, it causes an induction of rolling along the length axis and a CW path chirality. Together with ADP, a movement like that of sperm with intact membranes can be observed.

To demonstrate that the observed changes in 4D movements caused specifically by cAMP and are not an unspecific effect of all cyclic nucleotides in general, we used cGMP as a negative control. An induction of sperm motility in buffer containing cGMP could not be detected, illustrating the specific effect of cAMP. Beside flagellar movement and sperm velocity, rolling along the long axis was modified by different cAMP and ADP concentrations. The same cAMP (18 µM) and ADP (1 mM) concentrations necessary for flagellar movement were required for rolling of membrane intact sperm.

In earlier studies, we showed that non-capacitated as well as capacitated murine sperm have a conserved CW path chirality and proposed, that this swimming pattern is necessary for reaching the oocyte [21,39,43]. We further postulate that substantial changes in the intracellular Ca^2+^ concentration result in a loss of chirality and speculate that this is an important requirement to finalize fertilization [43]. In this study, we find that also a change in intracellular cAMP and ADP concentrations leads to a loss of path chirality in non-capacitated mouse sperm. To conclude: These 4D movement analysis indicates that defined cAMP, ADP, ATP and Mg^2+^ concentrations are necessary for stabilizing the beat cycle, velocity and path chirality of murine sperm.

### 3.2. Linear Swimming Depends on Alternating Chirality of Rolling

One proposed navigation mechanism needed for reaching the oocyte is the episodic rolling of mouse sperm along the long axis [38]. We already showed that alternating rolling is necessary for a linear swimming trajectory in mouse sperm and absence of rolling results in a circular swimming path [39]. Based on the hook-shaped mouse sperm head, the orientation of the cell can be described as Right-cheek-downmost (RCh) or Left-cheek-downmost (LCh). Sperm with LCh orientation show a swimming path circled CW and with RCh orientation the circular swimming path has a CCW direction [38,39]. In this study sea urchin sperm with a cone shaped head [49] were analyzed to identify the impact of sperm head morphology on the rolling and linear swimming behavior in 4D. We show that sea urchin sperm have a planar circular trajectory which is in line with the literature [33,63,64]. It is proposed that this swimming pattern is needed for the navigation in a chemical gradient [65], guiding the sperm to the oocyte, which in turn is necessary for successful fertilization.

Like in mouse sperm, the 3D flagellar movement is characterized by *Z*-plane excursions which travel as waves down the flagellum. However, sea urchin sperm exert smaller flagellar excursions in the *XY*-and *Z*-plane which is in line with non-rolling mouse sperm swimming in circles [39]. In addition, we were able to show by intensity measurements using DHM that sea urchins do not roll along their longitudinal axis. This result could be verified by using latex beads which are attached to the sperm head. The study of Jikeli and coworkers focused on the trajectory of sea urchin sperm, showing that sea urchin sperm swim along a helix and the head rotates around this helix axis [65]. By dealing with rolling along the sperm axis, our results do not conflict with the study of Jikeli. Based on our results we hypothesize that a symmetrical sperm head is not necessary for linear swimming trajectories and that in contrast linear swimming depends on alternating chirality of rolling along the sperm axis.

### 3.3. CABYR Maintains Conserved Path Chirality

Axonemes of invertebrates lack stabilizing structures like the FS or ODFs [44] and these sperm show no rolling along the long axis, so that it is likely that these structures are necessary for rolling. To examine if the CABYR protein, which is necessary for the organization of the FS [23] is essential for rolling, we analyzed sperm of the *Cabyr*-KO mice in comparison to sperm of wt animals. *Cabyr^−/−^* sperm show rolling along their long axis, indicating that CABYR is not necessary for rolling and therefore other or no proteins in the FS (for example, AKAP [66]) or ODF are essential for rolling.

CABYR knockout leads to an abnormal configuration of doublet microtubules proposing that a correct organization of the axoneme depends on the FS [23]. In addition, a defect in sperm motility, resulting in subfertility of *Cabyr^−/−^* male mice, could be detected [23]. The 4D movement analysis in the current study confirms these abnormalities in motility and extend them by identifying a decrease in the flagellar excursion in the *Z*-plane. In our recent work, we show a possible connection between the intracellular Ca^2+^ concentration and the swimming path chirality, by demonstrating that an alteration of the Ca^2+^ homeostasis after contact to ZP2 or by mutation of the CATSPER channel, the main Ca^2+^ channel in sperm flagellum [15,18,21], lead to a substantial change in path chirality [43]. CABYR is a calcium-binding protein localized in the principal piece, is phosphorylated during sperm capacitation and may play a role for capacitation-dependent phosphorylation in calcium signaling [45,67]. Interestingly, we also found a loss of the conserved CW path chirality in sperm lacking CABYR. Because loss of path chirality was already shown for *CatSper^−/−^* sperm [43], we hypothesize that the change in path chirality is caused by an imbalance of the intracellular Ca^2+^ homeostasis, A direct role of CABYR in Ca^2+^ signaling is unknown, so that further analysis for determining intracellular changes of Ca^2+^ ion concentration in *Cabyr^−/−^* sperm is needed.

In conclusion, this study shows that 4D sperm movement, especially path chirality, depends on the composition of the flagellum and on intracellular ion concentration. Changes in the architecture, for example, loss of the plasma membrane or FS, as well as changes in intracellular cAMP and ADP concentrations change the swimming behavior and lead to a loss of clockwise path chirality. This study provides new insights into the regulation of sperm motility and identifies CABYR as a necessary protein for maintaining CW path chirality. Based on our recent findings [43], we speculate that this sperm movement pattern is necessary for successful fertilization. The limitation of this work is based on the study of a small species diversity (mouse and sea urchin). In order to make general statements about sperm swimming behavior, the analyses applied in this work should also be carried out in other species such as humans and bulls. Increasingly detailed knowledge of the regulation of human sperm movement can improve the success rates of assisted reproduction and the diagnosis of male infertility.

## 4. Materials and Methods

### 4.1. Chemical

All chemicals were obtained from Sigma-Aldrich (Merck, Darmstadt, Germany) except PIPES ((Piperazine-N,N’-bis-(2-ethanesulphonic acid)), HEPES (N-2-Hydroxyethyl piperazine-N’-2-ethane sulphonic acid), EGTA (ethylene glycol bis(2-aminoethyl ether)-N,N,N’,N’-tetraacetic acid) and MgCl_2_ which were received from Carl Roth (Karlsruhe, Germany).

### 4.2. Animals

Wildtype NMRI mice were purchased from Charles River (Erkrath, Germany). The *Cabyr*-KO (Ca^2+^-binding tyrosine-phosphorylation-regulated protein) animals were purchased from RIKEN BioResource Research Center (Kyoto, Japan). The generation of *Cabyr*-KO mice was previously described [23]. Genotyping of these mice were performed with PCR analysis with the following primer: Cabyr forward 5′-AAGATATCTGTGCATTAGTAAGCAGTGG-3′ reverse 5′-AAGGATCCTGACGACCCTGCTGAAGT-GG-3′). Only wildtype and homozygous *Cabyr*-KO animals were used in this study. The mice were treated in accordance with guidelines approved by the University of Duisburg-Essen Animal Care and Use Committees (LANUV; protocol; AZ84-02.04.2014.A219).

Sea urchins of the species *Arbacia punctulata* were purchased from Aquatic Research Organism (ARO Inc., Hampton, NH, USA). They were housed in the Central Animal Facility of the University Clinic, University of Duisburg-Essen.

### 4.3. Sperm Preparation and Media

Murine sperm preparation was performed as described elsewhere [68]. In short, after preparation of the mouse, epididymidis and vas deferentia were transferred into a petri dish containing HS medium (in mM: 135 NaCl, 5 KCl, 1 MgCl2, 20 Hepes, 5 glucose, 10 DL-lactic acid and 10 pyruvic acid, pH 7.4), which was then incubated for 15 min at 37 °C and 5% CO_2_ for swim-out of sperm. After two centrifugation steps (5 min at 300× *g*) sperm were resuspended to a final concentration of 1–2 × 10^7^ cells/mL in HS medium and stored at room temperature. Sea urchins of the species *Arbacia punctulata* were obtained by injecting 0.5 M KCl in the body cavity. As a reaction to KCl, the sea urchins release sperm into their environment, here into the beaker filled with artificial sea water buffer ASW buffer (in mM: 423 NaCl, 9 KCl, 9.27 CaCl_2_, 22.9 MgCl_2_, 25.5 MgSO_4_, 10 HEPES and 0.1 EDTA, pH 7.8). The sperm were then pipetted out of the buffer and washed again with ASW buffer.

### 4.4. Demembranation and Reactivation of Sperm and Perfusion

In a petri dish, murine sperm of the stock solution (10 µL) were demembranated and reactivated in 3 mL of a demembranation and reactivation buffer (pH 7.8, in mM: 132 sucrose, 24 potassium glutamate, 20 Tris, 0.1% Triton X-100, 1 Dithiothreitol (DTT), 1 MgCl_2_, 0.5 EGTA, 1 ATP and 3 µM cAMP). The necessary amounts of ATP, ADP, cAMP and Mg^2+^ were added to the demembranation/reactivation buffer. The reactivated sperm were transferred to a FluoroDish^TM^ (World Precision Instruments, Friedberg, Germany) for local microperfusion or transferred to 100 µm deep chamber slides (Leja, Nieuw-Vennep, The Netherlands) for holographic imaging. All experiments were performed at room temperature.

### 4.5. Computer-Assisted-Sperm-Analysis (CASA)

Computer-Assisted-Sperm-Analysis (CASA) was performed with a MedeaLAB CASA System (v 5.5, Medical Technology GmBH, Altdorf, Germany) to determine 2D sperm motility parameters (total motility [%], proportion of fast and slow progressive sperm [%]). Murine sperm were washed and resuspended in HS buffer containing 5 mg/mL BSA (bovine serum albumin; Carl Roth GmbH, Karlsruhe, Germany). For analysis, 20 µL of the sperm suspension was loaded into a counting chamber (Makler, Sefi-Medical Instruments Ltd., Biosigma S.r.l., Cona (VE), Italy). Measurements were made on a minimum of 200 sperm from the recorded sperm samples.

### 4.6. Digital Holographic Microscopy (DHM)

Four-dimensional (4D) sperm motility analysis using digital holographic imaging is a method already established [39,43]. In short: For holographic records we used a transmission microscope with an off-axis configuration (type DHM^®^ T-1000) of Lyncée Tec (Lyncée Tec. SA, Lausanne, Switzerland) which operates in an off-axis mode. It is equipped with a 666 nm laser diodes source. For mouse sperm a Basler avA1000-100gm CCD camera and for sea urchin sperm a Basler aca1920-155um CCD camera (Basler AG, Ahrensburg, Germany) was used. Murine sperm were analyzed with a 20× objective, while sea urchin sperm were measured with a 63× objective. Open-source Spyder (Python 3.6.9) and Koala (V6, Lyncée Tec. SA, Waadt, Lausanne, Switzerland) software were used for offline processing of holographic data. *X*-, *Y*- and *Z*-coordinates of the heads of sperm were analyzed with Koala and custom-designed Spyder scripts. Calculating motility parameters such as the curvilinear velocity (VCL), lateral head displacement (ALH) were performed on *X*-, *Y*- and *Z*-values. Frame-by-frame tracing of the sperm flagella with Igor Pro^TM^ (Wavemetrics, Lake Oswego OR, USA) allowed to obtain *X*-, *Y*- and *Z*-coordinates like previously described [39,43]. The intensity of the center of sperm head was analyzed with the Manual Tracking plug-in of the MBF Collection for ImageJ (1.48 V, National Institute of Health, Washington, DC, USA) and was calculated relative to the cell-free background.

For 4D sperm motility analysis by DHM 1 × 10^6^ membrane intact murine sperm, as well as demembranated sperm incubated 5 min in HS-buffer added with specific ADP and cAMP concentrations and *Cabyr^−/−^* sperm were transferred to 100 μm deep chamber slides (Leja, Nieuw-Vennep, Netherlands) and analyzed using 20× magnification. Further, sea urchin sperm (1 × 10^7^ cells/mL) were transferred to a 100 µm deep chamber slide (Leja, Nieuw-Vennep, The Netherlands) and recording was performed by using a 63× magnification. For rolling analysis sperm were incubated for 5 min in ASW buffer containing 0.8 µm beads (Latex polystyrene beads, LB8, 0.8 µm mean particle size, Merck, Darmstadt, Germany) to allow unspecific binding to sperm heads.

### 4.7. Path Chirality Analysis Using Procrustes Alignments

The procedure of Procrustes analysis to determine path chirality of mouse sperm is described in depth elsewhere [39,43]. In short, the chirality of the sperm swimming path was determined by using sequences of roll-counter-roll cycles defined by rotation of the sperm head from right- (RCh) to left-cheek (LCh) and vice versa. After the use of different symmetry operations in IgorPro^TM^ and re-setting the first *XY*-coordinates of each roll cycle to the original position, the adjusted traces were averaged. The mean time derivative of theta (*dθ/dt*) (radian/s) were calculated, which allowed determination of path chirality. A clockwise (CW) path chirality is represented by a positive value whereas a negative value indicates a counter-clockwise (CCW) path chirality.

### 4.8. Sperm Beat Frequency and Waveform Analysis

The flagellar waveform was analyzed as previously described [30] with a Nikon Eclipse TE2000-U microscope. Images were collected at 300 Hz respectively by a M3 high speed camera (IDT, Tallahassee, FL, USA) using a 20× objective and the Motion-Studio 64 software (Imaging Solutions, Ehingen, Germany). For sperm beat frequency analysis, 10 µL of the sperm suspension in demembranation/reactivation buffer was transferred to a FluoroDish^TM^ (World Precision Instruments, Germany) and were allowed to adhere to the bottom during 3 Min incubation. Cells were perfused with different concentrations of ATP, ADP and Mg^2+^ for one minute (Figure 1B). Single sperm were selected for analysis with Image J (1.48V, National Institute of Health, Washington, DC, USA). Flagellar beat frequency was measured with semi-automated Igor Pro^TM^ software (Wavemetrics, Lake Oswego, OR, USA) like previously described [30,68].

### 4.9. Flagellar Movement and Trajectory Visualization in 3D

3D illustration of flagellar movement and swimming trajectory of sperm were performed with OriginPro 2020 (OriginLab Corporation, Northampton, MA, USA) by using *X*-, *Y*- and *Z*-coordinates of the head and the flagellar tracking. In general, values of one entire beat cycle were utilized and every fourth flagellar excursion was illustrated (for mouse sperm at 0, 40, …, 280 ms; for sea urchin sperm at 0, 15, …, 105 ms).

### 4.10. Simulation Methods

To model the swimming sperm cell, we describe the axoneme as a semiflexible, actively bending filament. Given the excellent agreement between simulation and experiment [69] we follow that approach, and activity is modeled as a simple propagating spontaneous curvature wave

C_0_(*s*) = *A*
*sin*(*ωt* − *ks*)

With spontaneous curvature amplitude *A*, beat frequency *ω*, and wavenumber *k*, ignoring second harmonic contributions. Here, we need to extend [69] to three dimensions, and thus discretize the Kirchoff model of semi-flexible filaments following [70] as octrahedal segments. Each segment consists of five beads at the corners of an octahedron held together by strong harmonic bonds of length $l_{0}=1$. The first and last bead of a segment always belong to two consecutive segments. The segments are further interconnected by (torsional) springs. The octahedral segments allow to define a local coordinate system at segment i, and in particular local curvatures and twist angles $\Omega_{1}^{i},\Omega_{2}^{i},\Omega_{3}^{i}$. The potential energy of the system than reads:

$$E_{\mathrm{bending}}=\frac{1}{2}\sum \kappa_{1}{\left(\Omega_{1}^{i}-\Omega_{1}^{i,*}\right)}^{2}+\kappa_{2}{\left(\Omega_{2}^{i}-\Omega_{2}^{i,*}\right)}^{2}+{\;\kappa }_{3}{\left(\Omega_{3}^{i}-\Omega_{3}^{i,*}\right)}^{2}$$

With bending energies $\kappa_{1}$ (in plane), $\kappa_{2}$ for out of plane bending, and $\kappa_{3}$ for torsion, and $\Omega_{?}^{i,*}$ corresponding spontaneous angles, stemming from the sperm activity $\Omega_{1}^{i,*}$= $C_{0}\left(s\right)\;l_{0}$. Hydrodynamic interactions are limited to anisotropic friction, i.e.:

$$\vec{v}=\zeta_{∥}^{-1}\;{\vec{F}}_{∥}+\zeta_{⊥}^{-1}\;{\vec{F}}_{⊥}$$

where $\zeta_{⊥}=2\;\zeta_{∥}$.

The head is modeled by increasing the friction of the first 10 beads of the filament by a factor of 4. The following 13 segments of the filament represent the mid-piece and thus no active torque is imposed. With a total of 150 segments consisting of 751 beads this corresponds approximately to a 45 µm long and 0.3 µm thick sperm, with a 3 µm long midpiece and a spherical head of 1.2 µm.

To give beads a chance to deform the flagellum, we model flexible sperm, by choosing a sperm number $Sp=\lambda {\left(\frac{5\zeta_{⊥}}{\kappa \;\tau_{b}}\right)}^{\frac{1}{4}}\sim 3\;$ [71] via $\;\tau_{b}=628$, *λ* = 94.5, and $\kappa_{1}=\kappa_{2}=\kappa_{3}=\kappa =15,625$.

Attachment of beads to the sperm cell is modeled as additional drag on the first two beads of the left side (looked from head to tail) of the flagellum. For modelling larger attached beads it would be more realistic to model the bead in more detail, however this is not the focus of this work. For the relevant bead diameters smaller than a few microns, this modelling approach is certainly useful. We expect at some bead diameter the sperm will start to show out of plane beating, however we did not observe this behavior so far, and leave this to a future work.

The restrictive force simulation employs a straightforward Euler integration with adaptive time stepping [72] where the change of the internal forces is considered as an error-estimate. Typical time steps are in the order of 5 × 10^−6^.

### 4.11. Transmission Electron Microscopy (TEM)

For standard TEM analysis, murine sperm (5 × 10^7^ cells/mL) were obtained as described under ‘sperm preparation and media’. Sperm of the suspension (2500 µL) were demembranated with the demembranation/reactivation buffer. Then two sperm solutions, one with control membranated sperm and one with demembranated sperm, were centrifuged for 5 min (300× *g*) and the pellet was washed twice in HS medium. Fixation was performed using 4% paraformaldehyde and 2% gluteralaldehyde in 0.1 M PHEM-buffer (60 mM PIPES, 25 mM HEPES, 10 mM EGTA and 2 mM MgCl_2_, 219 mOsm) (Carl Roth, Karlsruhe, Germany) for 1 h, followed by washing twice with PHEM-buffer (10 min at 2500× *g*). The pellet was embedded in 200 µL 4% agarose. After osmosis, samples were dehydrated with an ascending ethanol row (30%, 50%, 70%, 80% and 96%). Further, embedding of the samples occurred in EPON resin (Poly/Bed 812; Polysciences Europe GmbH, Hirschberg, Germany). EPON blocs were cut with a microtome (UC7, Leica, Wetzlar, Germany) to a thickness of 60 nm. TEM was performed on a LaB6 cathode-equipped Jeol 1400Plus (Tokio Akishima, Japan). The 16-bit TIFF image files were post-processed with Adobe^®^ Photoshop^®^ CS6 (Adobe Inc., San Jose, CA, USA).

### 4.12. Image Analysis

With the auto-adjustment tool of ImageJ images were reworked in contrast and brightness. The videos of the supplement were generated using the stack of cropped images in ImageJ and were stored in AVI format using JPEG compression.

### 4.13. Statistical Analysis

Creation of the graphics and statistical evaluation was performed with GraphPad Prism 9 (Statcon GmbH, Witzenhausen, Germany). Two-sided Student’s t-test were performed to compare two data sets, while ordinary one-way ANOVA were used to compare more than two datasets. Analysis were reported as means with S.E.M. or medians with interquartile ranges with N = number of independent measurements and n = number of determinations. Differences were classified as significant at *p* < 0.05.

## Data Availability

The data that support the findings of this study are available from the corresponding author upon reasonable request.

## Supplementary Materials

The following supporting information can be downloaded at: https://www.mdpi.com/article/10.3390/ijms231810607/s1.
